# The volatiles of pathogenic and nonpathogenic mycobacteria and related bacteria

**DOI:** 10.3762/bjoc.8.31

**Published:** 2012-02-22

**Authors:** Thorben Nawrath, Georgies F Mgode, Bart Weetjens, Stefan H E Kaufmann, Stefan Schulz

**Affiliations:** 1 Institut für Organische Chemie, Technische Universität Braunschweig, Hagenring 30, 38106 Braunschweig, Germany; 2 Department of Immunology, Max Planck Institute for Infection Biology, Charitéplatz 1, 10117 Berlin, Germany; 3 Pest Management Centre, Sokoine University of Agriculture, PO Box 3110, Chuo Kikuu, Morogoro, Tanzania; 4 Anti-Persoonmijnen Ontmijnende Product Ontwikelling (APOPO vzw), Groenenborgerlaan 171, 2020 Antwerpen, Belgium

**Keywords:** aromatic compounds, CLSA, terpenes, tuberculosis, volatile profile

## Abstract

Volatiles released by pathogenic and nonpathogenic mycobacteria, as well as by mycobacteria-related *Nocardia* spp., were analyzed. Bacteria were cultivated on solid and in liquid media, and headspace samples were collected at various times during the bacterial lifecycle to elucidate the conditions giving optimal volatile emission. Emitted volatiles were collected by using closed-loop stripping analysis (CLSA) and were analyzed by gas-chromatography–mass-spectrometry. A wide range of compounds was produced, although the absolute amount was small. Nevertheless, characteristic bouquets of compounds could be identified. Predominantly aromatic compounds and fatty-acid derivatives were released by pathogenic/nonpathogenic mycobacteria, while the two *Nocardia* spp. (*N. asteroides* and *N. africana*) emitted the sesquiterpene aciphyllene. Pathogenic *Mycobacterium tuberculosis* strains grown on agar plates produced a distinct bouquet with different volatiles, while liquid cultures produce less compounds but sometimes an earlier onset of volatile production because of their steeper growth curves under this conditions. This behavior differentiates *M. tuberculosis* from other mycobacteria, which generally produced fewer compounds in seemingly lower amounts. Knowledge of the production of volatiles by *M. tuberculosis* can facilitate the rational design of alternative and faster diagnostic measures for tuberculosis.

## Introduction

Tuberculosis (TB) remains one of the most threatening diseases on earth. In 2008, up to 2 million people died as a result of TB infection [1]. The causative agent, *M. tuberculosis,* has a highly flexible physiology and metabolism that allows it to adapt to changes in the environment during the course of an infection [2]. Other pathogenic mycobacteria cause diseases such as leprosy, evoked by *M. leprae*, or buruli ulcer, due to infection by *M. ulcerans* [3–4]. In addition, numerous nonpathogenic and facultative pathogenic mycobacteria exist.

Although several diagnostic measures have been developed for TB diagnosis [5–6], most of these techniques are expensive, e.g., immunological tests using antigens, DNA analysis, or specific culturing conditions, especially for developing countries with the highest burden of TB [7–9]. Thus, the analyses of species-specific volatiles obtained from breath samples of potentially infected individuals, or from the bacteria themselves, have recently been proposed for rapid diagnosis of TB [8–11]. The feasibility of these methods is supported by reports that trained *Cricetomys* rats can distinguish between sputum samples from TB-infected and noninfected persons [12–13].

**Figure 1:**
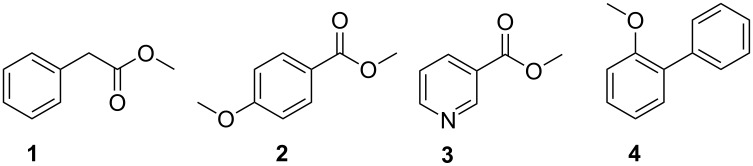
Volatile compounds released by *Mycobacterium tuberculosis* and *M. bovis* identified in previous studies [9].

While the first studies based on electronic sensors or fuzzy-logic experiments gave promising results [8,10], recent studies have focused on the characteristic compounds released by pathogenic *M. bovis* and *M. tuberculosis* strains [9,11]. Compounds from these species were also expected to occur in breath samples from TB patients [8,10]. In these studies, volatiles were collected by using solid-phase micro-extraction (SPME). Methyl phenylacetate (**1**), methyl *p*-anisate (**2**), methyl nicotinate (**3**), and *o*-phenylanisole (**4**) were emitted by pathogenic *M. bovis* and *M. tuberculosis* (Figure 1), while nontuberculous mycobacteria did not produce these four compounds [9]. Due to a central role in mycobacterial metabolism [14], nicotinic acid, closely related to (**3**), was used in these studies as a reference compound from the breath of TB-positive patients [11]. Infected individuals also released nicotinic acid (excluding smoking patients in this study), proving the utility of this approach [11].

Nevertheless, a broad analysis of the volatiles produced by *M. tuberculosis*, nontuberculous mycobacteria and mycobacteria-related species has not been performed. This knowledge is of utmost importance in the identification of potential compounds for use in pattern-recognition methods, such as electronic noses and trained *Cricetomys* rats. We report here on the identification of volatiles produced by different strains of *M. tuberculosis*, as well as of *M. smegmatis*, *M. aurum*, *M. neoaurum*, *M. aichiense*, *M. scrofulaceum*, *M. avium* spp. *avium*, *M. vaccae*, *Nocardia africana*, and *N. asteroides*, using closed-loop stripping analysis (CLSA) for the collection of volatile compounds [15–16]. This method allows sampling for longer periods than SPME, and usually results in a lower detection limit. Compounds not detectable by SPME can thus be detected. SPME preferentially favors less volatile compounds [17]. Furthermore, SPME may have a discriminative effect, and our experience has shown that minor components are often not detected when large amounts of a major component are present.

The investigated mycobacteria were in different stages of their lifecycles and grown on various different media. The volatiles released under these conditions were identified and their formation under different conditions is discussed.

## Results and Discussion

Volatiles released from different mycobacteria and *Nocardia* spp. grown on solid or in liquid media were collected by using CLSA as described previously [15–16]. The headspace extracts were analyzed by gas-chromatography–mass-spectrometry (GC–MS). Compound identification was performed by comparison of mass spectra and gas-chromatographic retention indices with those of authentic reference compounds and mass-spectra libraries [18].

### Analysis of bacteria grown on a solid medium

**Figure 2:**
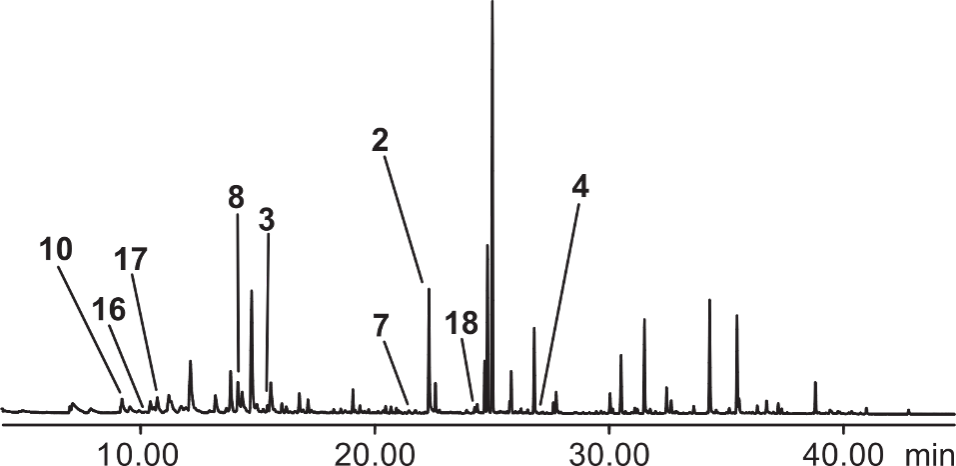
Total ion chromatogram of a headspace extract of a culture of *Mycobacterium tuberculosis* strain 2, grown on a 7H11 solid medium. Nonlabeled peaks originate from the nutrient medium.

Different strains of *M. tuberculosis* grown on a 7H11 solid medium were analyzed after different incubation periods (days). The detection of bacteria-specific compounds was assured by the analysis of headspace samples from a sterile medium incubated in parallel with bacterial strains. Although many compounds were released from the medium, bacteria-specific compounds were distinguishable. A typical gas chromatogram is shown in Figure 2, revealing that many compounds are formed from the nutrient medium that is necessary to grow the bacteria. The identified compounds are listed in Table 1.

**Table 1:** Volatiles released by *Mycobacterium tuberculosis* grown on a 7H11 solid medium.^a^

Compound	*I*	strain 1	strain 2	strain 5	strain 6	H37Ra

4-Hydroxy-4-methylpentan-2-one (**14**)	847	– – – ^b^	– – – + ^c^	– + – – – – – ^d^	– – – ^e^	– ^f^
4-Pentanolide (**10**)	950	+ + +	+ + + +	– – + + + + +	+ + +	+
Methyl 2-furoate (**15**)	978	– – –	+ + – +	– – + + – – –	+ + –	+
3-Methyl-4-pentanolide (**16**)^g^	984	– + +	+ + + +	– – – – – – –	– – –	–
Unknown	1006	– – +	+ – + –	– – + + + + +	– – +	–
Unknown	1021	+ + –	+ + – +	– – – – – – –	– – –	–
4-Methylanisol (**5**)	1021	– – +	– – + –	– – + + + + +	– – +	–
Unknown	1084	– + +	+ + + –	– – + + + + +	– + +	–
Methyl benzoate (**8**)	1093	+ + +	+ + + +	+ + + + + + +	+ + +	+
2-Phenylethanol^h^	1113	+ + +	+ + + +	– + + + + + +	+ + +	–
4-Methyl-5-hexanolide (**17**)^g^	1133	– – –	– + + –	– – – – – – –	– – –	–
Methyl nicotinate^c^ (**3**)	1136	+ + +	+ + + +	– + + + + + +	+ + +	–
Ethyl benzoate (**9**)	1169	– – –	– – – –	– – – + + + +	– – –	+
Methyl phenylacetate (**1**)	1177	+ + –	– + + +	– – – + + + +	+ + +	–
Methyl salicylate (**6**)	1193	+ – –	– – – –	– – + – – – +	– + –	+
Benzothiazole	1222	– – –	– – – +	– – – + – – –	– – –	–
Methyl 2-aminobenzoate (**7**)	1341	– – –	+ + – –	– – – + – – –	– – –	–
Methyl *p*-anisate (**2**)	1376	+ + +	+ + + +	– + + + + + +	+ + +	–
Ethyl *p*-anisate (**18**)	1450	– + –	+ + – –	– + + + – + +	+ + –	–
2-Phenylanisol (**4**)	1559	+ + +	+ + + +	– + + + + + +	+ + +	–

^a^*I*: gas-chromatographic retention index on DB-5; +: compound detected in sample; –: compound not detected in sample. ^b^The results of three analyses are shown for each experiment performed on differently aged cultures: 25 days (d), 26 d, 32 d. ^c^The results of four analyses are shown for each experiment performed on differently aged cultures: 20 d, 22 d, 30 d, 33 d. ^d^The results of seven analyses are shown for each experiment performed on differently aged cultures: 4 d, 9 d, 18 d, 21 d, 28 d, 29 d, 48 d. ^e^The results of three analyses are shown for each experiment performed on differently aged cultures: 19 d, 23 d, 31 d. ^f^The results of an analysis on a 14 d old culture are shown. ^g^Mixture of both diastereomers; *I* of first eluting isomer is shown. ^h^Traces present in the medium.

**Figure 3:**
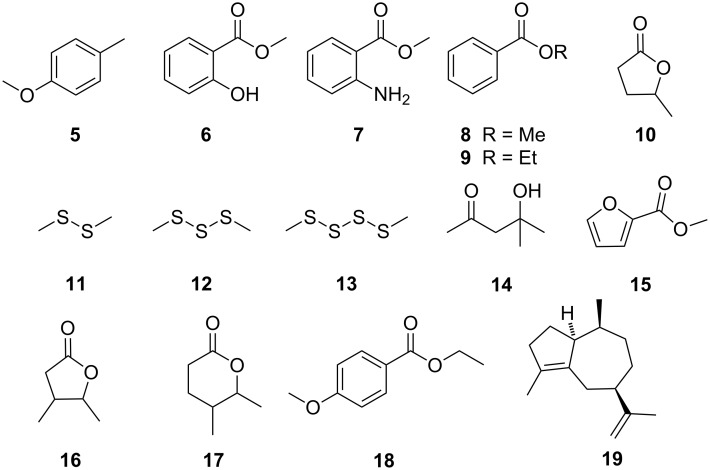
Volatiles released by mycobacteria and *Nocardia* spp. grown on a 7H11 solid medium.

In addition to previously reported compounds **1**–**4** [9], several new volatiles were identified, predominantly aromatic compounds, such as 4-methylanisole (**5**), methyl salicylate (**6**), methyl 2-aminobenzoate (**7**), and methyl and ethyl benzoate (**8** and **9**), as well as fatty-acid derivatives, for example, 4-pentanolide (**10**) (Figure 3). Many of the compounds listed in Table 1 are known volatiles from other bacteria [19].

As shown in Table 1, early cultures produced few compounds; only those older than 18 days produced a bouquet of compounds. This is probably due to the slow growth of many mycobacteria [7,20]. Increased production of volatile compounds occurred in the stationary phase (i.e., starting after three weeks of growth), a phenomenon that corroborates the recent findings that *M. tuberculosis* culture is detected by trained rats mostly at this growth stage [20]. A full description of the growth stages used in this report has recently been published [20]. The analyses of cultures of *M. smegmatis*, *M. aurum*, *M. neoaurum*, *M. aichiense*, *M. scrofulaceum*, *M. avium* ssp. *avium*, and *M. vaccae* revealed that these diverse nontuberculous mycobacteria did not produce as many different compounds as *M. tuberculosis* (Table 2), and what they did produced was often in lower quantities. The exceptions are *M. scrofulaceum* and *M. avium* ssp. *avium*, which cause cervical lymphadenitis in children and avian TB, as well as opportunistic infections in immunocompromised humans [21–22].

**Table 2:** Volatiles released by diverse nontuberculous mycobacteria grown on a 7H11 solid medium.^a^

Compound	*I*	Msm	Mau	Mn	Mai	Msc	Maa	Mv

Dimethyl disulfide (**11**)		– – –^b^	+ + –^c^	+ + +^d^	– – –^e^	– –^f^	– –^g^	– –^h^
4-Pentanolide (**10**)	950	+ + +	+ + +	+ + +	+ + +	+ +	+ +	– –
Dimethyl trisulfide (**12**)	969	– + –	+ + –	+ + +	– – –	– –	– –	– –
Methyl 2-furoate (**15**)	978	– – –	– – –	– – –	– – –	+ +	– –	– –
3-Methyl-4-pentanolide (**16**)^i^	984	– – –	– – –	– – –	– – –	– –	– +	– –
6-Methylhept-5-en-2-one	988	– – –	– – –	– – –	– – –	– –	+ +	– –
Unknown	1006	– – –	– – –	– – –	– – –	+ +	– +	– –
Methyl benzoate (**8**)	1093	– – –	– – –	– – –	– – –	+ +	– –	– –
2-Phenylethanol^j^	1111	+ + –	– – –	– ––	– – –	+ +	+ +	– –
Ethyl benzoate (**9**)	1169	– – –	– – –	– – –	– – –	+ +	– –	– –
Methyl phenylacetate (**1**)	1177	– – –	– – –	– – –	– – –	+ +	+ –	– –
Methyl salicylate (**6**)	1193	– – –	– – –	– – –	– – –	+ +	–+	– –
Dimethyl tetrasulfide (**13**)	1206	– – –	– – –	+ + +	– – –	– –	– –	– –
Ethyl salicylate	1270	– – –	– – –	– – –	– – –	+ +	– –	– –
Methyl methylsalicylate	1315	– – –	– – –	– – –	– ––	– –	+ +	+ +
Unknown sesquiterpenes		– – –	– – +	– – –	+ + +	– –	– –	+ +

^a^*I*: gas-chromatographic retention index on DB-5; +: compound detected in sample; –: compound not detected in sample. Msm: *M. smegmatis,* Mau: *M. aurum*, Mn: *M. neoaurum,* Mai: *M. aichiense,* Msc: *M. scrofulaceum,* Maa: *M. avium* ssp. *avium,* Mv: *M. vaccae.* ^b^The results of three analyses are shown for each experiment performed on differently aged cultures: 2 d, 3 d, 4 d. ^c^The results of three analyses are shown for each experiment performed on differently aged cultures: 4 d, 5 d, 11 d. ^d^The results of three analyses are shown for each experiment performed on differently aged cultures: 5 d, 6 d, 13 d. ^e^The results of three analyses are shown for each experiment performed on differently aged cultures: 3 d, 8 d, 9 d. ^f^The results of two analysis are shown for each experiment performed on differently aged cultures: 6 d, 11 d. ^g^The results of two analysis are shown for each experiment performed on differently aged cultures: 5 d, 11 d. ^h^The results of two analysis are shown for each experiment performed on differently aged cultures: 4 d, 7 d. ^i^*I* of first eluting isomer is shown. ^j^Traces present in the medium.

Although certain volatile, aromatic compounds, such as **1**, **6**, **8**, and **9**, were produced in some cases, many compounds from extracts of *M. tuberculosis* were absent in the other mycobacteria. The low emission rate of volatile compounds was not a result of the early stage at which the mycobacteria were analyzed, since all strains were fast-growing [20–21,23–28]. Interestingly, some of the bacteria emitted sulfur-containing compounds, such as dimethyl disulfide (**11**), dimethyl trisulfide (**12**), and dimethyl tetrasulfide (**13**), which are known bacterial volatiles [19]; these compounds were not emitted from *M. tuberculosis*.

While the mycobacteria grown on the 7H11 solid medium emitted a wide variety of volatiles, *Nocardia asteroides* produced, even in different ages, only 4-pentanolide (**10**) (Table 3). Additionally, *N. africana* also released only one compound, the sesquiterpene aciphyllene (**19**), which is a known volatile from several plants [29], and the endophytic fungus *Muscodor albus* [30]. The structure of **19** has recently been revised [29]. This compound may act as marker for norcardiae, as it was produced as a single compound by *Nocardia* spp. grown on a 7H11 medium and has so far not been reported for other bacteria [19].

**Table 3:** Compounds released by *Nocardia* spp. grown on a 7H11 solid medium.^a^

Compound	*I*	*N. asteroides*				*N. africana*		
				
		5 d	16 d	18 d		3 d	6 d	10 d

4-Pentanolide (**10**)	950	+	+	+		–	–	–
Aciphyllene (**19**)	1499	–	–	–		+	+	+

^a^*I*: gas-chromatographic retention index on DB-5; +: compound detected in sample; –: compound not detected in sample; d: days.

### Analysis of bacteria grown in diverse liquid media

**Figure 4:**
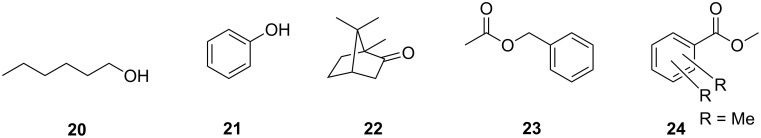
Volatiles released by *M. tuberculosis* grown in a 7H9 broth liquid medium.

In these experiments, two different media were used for culturing bacteria. *Nocardia* spp. were only cultured on the 7H9 broth medium, and all mycobacteria were grown on the 7H9 broth and the Sauton liquid media. The results of the analyses of different strains of *M. tuberculosis* grown on the 7H9 broth medium are shown in Figure 4 and Table 4.

**Table 4:** Volatiles released by *Mycobacterium tuberculosis* grown in a 7H9 broth medium.^a^

Compound	*I*	Strain 2			Strain 5					Strain 6	
						
		12 d	33 d		3 d	6 d	10 d	13 d		14 d	15 d

1-Hexanol (**20**)	890	–	–		+	–	–	–		–	–
4-Pentanolide (**10**)	950	+	+		–	+	+	–		+	–
Methyl butenolide	979	–	+		–	+	–	–		–	–
Phenol (**21**)	984	–	+		–		–	–		–	–
Unknown	1021	–	+		+	+	–	–		–	–
4-Methylanisol (**5**)	1021	–	+		–	+	–	–		–	–
Methyl benzoate (**8**)	1093	–	+		–	+	–	+		+	+
2-Phenylethanol^b^	1113	+	–		–	+	+	+		+	+
Methyl nicotinate (**3**)^b^	1136	–	–		–	+	–	–		–	+
Camphor (**22**)	1143	–	–		+	+	–	–		–	+
Benzyl acetate (**23**)	1164	–	+		–		–	–		–	–
Methyl phenylacetate (**1**)	1177	–	–		–	+	–	–		+	+
Benzothiazole	1222	–	+		–	–	–	–		–	–
Methyl 2-aminobenzoate (**7**)	1341	–	+		–	–	–	–		–	–
Methyl dimethylbenzoate (**24**)	1348	–	+		–	–	–	–		+	–

^a^*I*: gas-chromatographic retention index on DB-5; +: compound detected in sample; –: compound not detected in sample; d: days. ^b^Traces present in the medium.

A 33-day-old culture of *M. tuberculosis* strain 2, and a 6-day-old culture of strain 5 produced the largest variety of compounds. Again, aromatic compounds including the previously observed volatiles **1**, **5**, **7**, and **8**, as well as the fatty acid derivative **10**, were present in the headspace extracts.

Frequently, mycobacteria are observed to grow faster in liquid medium than on solid medium [31–32]. Consistent with this, *M. tuberculosis* strain 5 produced the most volatiles after six days of growth, unlike cultures of the same age grown on solid medium. Heavy inoculation of a fresh medium with a liquid culture of *M. tuberculosis* at the exponential growth phase may also generate a high yield of volatiles. Several volatiles released in the solid-medium experiments were not observed in liquid culture, and vice versa. On a solid medium, compounds **14**–**18** were formed but were not emitted in liquid cultures. In contrast, liquid cultures of *M. tuberculosis* released 1-hexanol (**20**), phenol (**21**), camphor (**22**), benzyl acetate (**23**), methyl dimethylbenzoate (**24**), and a methyl butenolide, all of which were absent in strains cultured on a solid medium.

The headspace extracts of the nontuberculous mycobacteria cultured in the 7H9 broth medium were investigated and the results (Table 5) revealed that most of the nontuberculous mycobacteria produced only a few volatiles, with the exception of *M. scrofulaceum* and *M. avium* ssp. *avium*, which produced a larger variety of compounds, including various aromatic volatiles also emitted by *M. tuberculosis*.

**Table 5:** Volatiles released by diverse nontuberculous mycobacteria grown in the 7H9 broth medium.^a^

Compound	*I*	Msm	Mau	Mn			Mai		Msc	Maa	Mv
									
		8 d	6 d	7 d	13 d		5 d	14 d	9 d	10 d	8 d

2-Methylbutanol		–	–	–	–		–	–	+	–	–
Methyl isovalerate		–	–	–	–		–	–	+	–	–
1-Hexanol (**20**)	890	+	–	–	–		–	–	–	–	–
Isobutyl isobutyrate	919	–	–	–	–		–	–	+	–	–
4-Pentanolide (**10**)	950	+	–	–	–		–	–	–	+	+
Methyl butenolide	979		+	+	+		+	+	–	–	–
3-Methyl-4-pentanolide (**16**)^b^	984	–	–	–	–		–	–	–	–	+
Methyl benzoate (**8**)	1093	–	–	–	–		–	–	+	+	–
2-Phenylethanol^c^	1111	+	–	–	–		–	–	+	+	+
Camphor (**22**)	1143	–	–	–	–		–	–	–	–	+
Methyl phenylacetate (**1**)	1177	–	–	–	–		–	–	+	+	–
Methyl salicylate (**6**)	1193	–	–	–	–		–	–	+	+	+
Indole	1289	–	–	–	–		+	+	–	–	–
2-Aminoacetophenone	1299	+	–	–	–		–	–	–	–	–
Methyl dimethylbenzoate (**24**)	1348	–	–	–	–		–	–	+	+	–

^a^*I*: gas-chromatographic retention index on DB-5; +: compound detected in sample; –: compound not detected in sample; d: days; Msm: *M. smegmatis,* Mau: *M. aurum*, Mn: *M. neoaurum,* Mai: *M. aichiense,* Msc: *M. scrofulaceum,* Maa: *M. avium* ssp. *avium,* Mv: *M. vaccae*. ^b^Mixture of both diastereomers. ^c^Traces present in the medium.

The analyses of the *Nocardia* strains revealed interesting differences between the two media types. Although aciphyllene (**19**) was again produced by *N. africana*, it was also present in the bouquet of an early culture of *N. asteroides* (Table 6). Additionally, both bacteria produced several unknown diterpenoids. Such relatively large compounds have rarely been reported as bacterial volatiles [33–34].

**Table 6:** Volatiles released by *Nocardia asteroides* and *N. africana* grown in the 7H9 broth medium.^a^

Compound	*I*	*N. asteroides*			*N. africana*		
				
		4 d	14 d		2 d	5 d	11 d

Aciphyllene (**19**)	1499	+	–		+	+	+
Unknown diterpenoids		+	–		+	–	+

^a^*I*: gas-chromatographic retention index on DB-5; +: compound detected in sample; –: compound not detected in sample; d: days.

In additional experiments, a Sauton liquid medium was used to culture *M. tuberculosis* (Table 7). The two strains of *M. tuberculosis* produced fewer volatiles compared to cultures on the solid medium of the same age. The growth of *M. tuberculosis* in the Sauton liquid medium was slower than in other liquid media. Thus, the type of medium used influenced the production of volatiles. Few volatiles were produced, including predominantly aromatic compounds, such as **1**, **2**, **8**, and **24**, especially in the 46-day-old culture of *M. tuberculosis* strain 5.

**Table 7:** Volatiles released by *Mycobacterium tuberculosis* grown in a Sauton liquid medium.^a^

Compound	*I*	Strain 5				Strain 6
				
		24 d	26 d	46 d		25 d

2-Hydroxypentan-3-one		–	–	–		+
Trimethyloxazole		–	+	+		+
Unknown	947	–	–	+		+
Benzylalcohol	1035	–	+	–		+
Phenylacetaldehyde	1041	–	–	–		+
Methyl benzoate (**8**)	1093	–	–	+		–
2-Phenylethanol^b^	1113	+	+	+		+
Methyl phenylacetate (**1**)	1177	–	–	+		–
Methyl *p*-anisate (**2**)	1376	–	–	+		–
Methyl dimethylbenzoate (**24**)	1348	–	–	+		–

^a^*I*: gas-chromatographic retention index on DB-5; +: compound detected in sample; –: compound not detected in sample; d: days. ^b^Traces present in the medium.

Analyses of the headspace extracts from nontuberculous mycobacteria grown in the Sauton medium (*M. smegmatis*, *M. aurum*, *M. neoaurum*, *M. aichiense*, and *M. avium* ssp. *avium*) showed that fewer compounds were produced by these species in this medium (Table 8).

**Table 8:** Volatiles released by diverse nontuberculous mycobacteria grown in a Sauton liquid medium.^a^

Compound		Msm	Mau	Mn	Mai	Maa

2-Hydroxypentan-3-one		– – –^b^	– – – – ^c^	– – – – ^d^	– – – ^e^	+ ^f^
Benzylalcohol	1035	+ + –	– – – –	+ + – –	– – +	+
Methyl benzoate (**8**)	1093	– – –	– – – –	– – – –	– – –	+
Linalool	1099	– – –	– – – –	+ – – –	+ – –	+
2-Phenylethanol^g^	1113	+ + +	– – – –	+ + – –	– – –	+
Methyl phenylacetate (**1**)	1177	– – –	– – – –	– – – –	– – –	+
Methyl dimethylbenzoate (**24**)	1348	– – –	– – – –	– – – –	– – –	+
Unknown sesquiterpenes		+ + –	– – – –	– – – –	– – –	+

^a^*I*: gas-chromatographic retention index on DB-5; +: compound detected in sample; –: compound not detected in sample. Msm: *M. smegmatis,* Mau: *M. aurum*, Mn: *M. neoaurum,* Mai: *M. aichiense,* Maa: *M. avium* ssp. *avium. *^b^The results of three analyses are shown for each experiment performed on differently aged cultures: 7 d, 8 d, 9 d. ^c^The results of four analyses are shown for each experiment performed on differently aged cultures: 1 d, 7 d, 11 d, 12 d. ^d^The results of four analyses are shown for each experiment performed on differently aged cultures: 5 d, 6 d, 10 d, 20 d. ^e^The results of three analyses are shown for each experiment performed on differently aged cultures: 8 d, 9 d, 10 d. ^f^The results of an analysis on day 10 is shown. ^g^Traces present in the medium.

As observed for the 7H9 medium, only *M. avium* ssp. *avium* emitted a diverse array of volatiles, while the other mycobacteria produced a few compounds only. The results of different analyses showed that *M. tuberculosis* produces more compounds than the previously described volatiles **1**–**4**, especially on the solid medium.

Apart from the production of specific compounds such as **3** and **4**, *M. tuberculosis* was characterized by a more pronounced production of volatiles compared to other mycobacteria and associated *Nocardia* bacteria. While many of these compounds, such as 2-phenylethanol, **1**, or **8**, are commonly found in some, but not all, bacteria [19], the individual components contribute to a specific bouquet of volatiles. This bouquet probably enables olfactory detection of *M. tuberculosis* by trained *Cricetomys* rats [13], or could enable potential detection by electronic noses. Only *M. scrofulaceum* and *M. avium* ssp. *avium* were found to produce a nutrient mixture of volatiles while other strains did not. The volatiles identified belonged predominantly to the biosynthetic class of aromatic compounds, while metabolites of the fatty-acid-biosynthesis pathway were also present.

Since *M. tuberculosis* grown in the Sauton liquid medium produced only a few volatiles after more than 25 days, these experimental conditions are suboptimal. *M. tuberculosis* cultivated in the 7H9 broth medium emitted after a shorter incubation time more volatiles in comparison to cultures grown in solid medium. However, the number of volatiles produced compared to nontuberculous mycobacteria was much lower than for mycobacteria grown on solid medium. The compounds emitted by *M. tuberculosis* grown on the 7H9 broth medium are known, except for **3**, but are also released from other bacteria, thus diminishing their diagnostic potential [19,35–36]. Compound **19** may play a role as a marker for different *Nocardia* strains, depending on growth conditions. Further studies should focus on enhancing the production of volatiles in liquid medium, which support the rapid growth of bacteria. Currently the identified volatile compounds produced by *M. tuberculosis* are tested for tuberculosis detection by using *Cricetomys* rats.

## Conclusion

In conclusion, profound qualitative and quantitative differences (number of compounds as well as probably higher emission rates) in the bouquet of volatiles from *M. tuberculosis*, nontuberculous mycobacteria and other bacteria grown on different media were found. *M. tuberculosis* produces a distinctive bouquet of compounds, consisting of compounds known to be produced by other bacteria, but also including relatively specific compounds such as methyl nicotinate (**3**). Variations occurred within individual analyses and also between different media. Therefore, it seems unlikely that GC–MS analyses of individual cultures can be used as a diagnostic tool. Nevertheless, a system able to detect mixtures within a given compositional tolerance seems more promising. In this sense, trained *Cricetomys* rats seem to be well suited for detection [20]. In a more technical variant, experiments with electronic noses, able to discriminate between different odor profiles, seem to be more promising for the detection of *Mtb*-specific odor profiles and, thus, could be potentially used for TB diagnosis.

## Experimental

### Media and growth conditions

Bacterial colonies from a 7H11 solid medium (Becton, Dickinson & Co., Sparks, USA, see Supporting Information File 1) or bacteria culture (100 µL) from a 7H9 liquid medium (Becton, Dickinson & Co., Sparks, USA, see Supporting Information File 1) were inoculated on a 7H11 solid medium and were spread out with sterile disposable loops to cover the entire plate/medium surface. Plates were wrapped in parafilm and aerobically incubated at 28–37 °C.

For bacteria grown in the 7H9 liquid medium, the inoculum (500 µL) was aseptically transferred from actively growing cultures and inoculated into fresh medium (15–20 mL). Cultures were incubated at 28–37 °C under aerobic conditions.

Bacterial cultures for inoculation into Sauton medium (without glycerol) were washed in order to remove traces of ingredients from the stock-culture medium (7H9). Washing was done three times by mixing the culture (3 mL) with phosphate-buffered saline (PBS; 10 mL) and centrifuging at 4000 rpm for 10 min. Supernatants were decanted and PBS (10 mL) was added to the pellet, which was dissolved by careful pipetting. The suspension was centrifuged again at 4000 rpm for 10 min. The final pellet was suspended in sterile PBS (4 mL), mixed thoroughly and 500 µL of the suspension was subcultured into fresh Sauton medium (30 mL). The Sauton medium consisted of the following ingredients in 1000 mL of distilled water: asparagine (4 g), MgSO_4_ (0.5 g), K_2_PO_4_ (0.5 g), citric acid (1.83 g), ferric ammonium citrate (0.05 g), D-(+)-glucose monohydrate (4.82 g), and sodium pyruvate (4.82 g). The pH was adjusted to 6.8 and the medium was filter-sterilized by using a 0.22 µm membrane filter (Millipore Corp., USA). Cultures were incubated at 37 °C aerobically.

### Sampling of volatiles

Volatile organic compounds emitted by cell cultures of the different mycobacteria were collected by using the CLSA technique [14–15]. The volatiles were adsorbed on charcoal (Chromtech; Precision Charcoal Filter, 5 mg) for 24 h, and then eluted with 30 µL of CH_2_Cl_2_. The obtained extracts were immediately analyzed by GC–MS, and stored at –30 °C.

### GC–EIMS analysis

GC–EIMS analyses of the samples were carried out on a HP-6890 GC system connected to a HP-5973 mass-selective detector fitted with a BPX5 fused-silica capillary column (25 m, 0.22 mm i.d., 0.25 µm film; SGE, Australia), or on an Agilent 7890A connected with an Agilent 5975C inert mass detector fitted with a HP-5MS fused silica capillary column (30 m, 0.25 mm i.d., 0.25 μm film; J&W Scientific, USA). Conditions for the HP-6890/HP-5973 system were as follows: Inlet pressure: 77.1 kPa, 23.3 mL He min^–1^; injection volume: 1 µL; transfer line: 300 °C; electron energy: 70 eV. The GC was programmed as follows: 5 min at 50 °C, increasing at 5 °C min^–1^ to 320 °C, operating in splitless mode (60 s valve time). Conditions for the Agilent 7890A/Agilent 5975C system were as follows: Inlet pressure: 77.1 kPa, He 23.3 mL min^–1^; injection volume: 1 µL; transfer line: 300 °C; electron energy: 70 eV. The GC was programmed as follows: 5 min at 50 °C, increasing at 5 °C min^–1^ to 320 °C, operated in splitless mode (60 s valve time); He carrier gas at 1 mL min^–1^ (HP-6890) or 1.2 mL min^−1^ (Agilent 7890A).

Retention indices (*I*) were determined from a homologous series of *n*-alkanes (C8–C35) [19]. Identification of compounds was performed by comparison of mass spectra to the Wiley-6 Library, NIST 07, and the Essential Oils Library (Massfinder) and gas chromatographic retention indices, as well as by comparison with synthetic samples. Details can be found in the supporting information. The relative emission of volatiles was roughly estimated from peak areas, although overlapping peaks from the medium and the known difficulty in using CLSA for quantification allowed for only a rough approximation.

## Supporting Information

File 1 Identification of compounds.
