# Supplementary material for: The volatiles of pathogenic and nonpathogenic mycobacteria and related bacteria
Source: Beilstein J Org Chem. 2012 Feb 22;8:290–9. doi: 10.3762/bjoc.8.31 (PMC3302092; doi:10.3762/bjoc.8.31)
Supplement: File 1 — Identification of compounds. [file Beilstein_J_Org_Chem-08-290-s001.pdf]

# Supporting Information

## for

# The volatiles of pathogenic and nonpathogenic mycobacteria and related bacteria

Thorben Nawrath[1], Georgies F. Mgode[2,3], Bart Weetjens[4], Stefan H. E. Kaufmann[2] and Stefan Schulz[1*]

Address: [1]Institut für Organische Chemie, Technische Universität Braunschweig, Hagenring 30, 38106 Braunschweig, Germany, [2]Department of Immunology, Max Planck Institute for Infection Biology, Charitéplatz 1, 10117 Berlin, Germany, [3]Pest Management Centre, Sokoine University of Agriculture, PO Box 3110, Chuo Kikuu, Morogoro, Tanzania and [4]Anti-Persoonmijnen Ontmijnende Product Ontwikelling (APOPO vzw), Groenenborgerlaan 171, 2020 Antwerpen, Belgium

Email: Stefan Schulz* - stefan.schulz@tu-bs.de

*Corresponding author

## Identification of compounds

The following compounds were identified by comparison with mass spectra and retention indices of authentic samples: 4-hydroxy-4-methylpentan-2-one (**14**), 4-pentanolide (**10**), methyl 2-furoate (**15**), 4-methylanisol (**5**), methyl benzoate (**8**), 2-phenylethanol, methyl nicotinate (**3**), ethyl benzoate (**9**), methyl phenylacetate (**1**), methyl salicylate (**6**), benzothiazole, methyl 2-aminobenzoate (**7**), methyl *p*-anisate (**2**), ethyl *p*-anisate (**18**), 2-phenylanisol (**4**), dimethyl disulfide (**11**), dimethyl trisulfide (**12**), 6-methylhept-5-en-2-one, dimethyl tetrasulfide (**13**), ethyl salicylate, 1-hexanol (**20**), phenol (**21**), camphor (**22**), 2-methylbutanol, methyl isovalerate, isobutyl isobutyrate, indole, 2-aminoacetophenone, 2-hydroxypentan-3-one, linalool. These compounds were available in our in-house compound library, originating from suppliers such as Aldrich, Acros, Fluka or, were synthesized previously.

The lactones 3-methyl-4-pentanolide (**16**) and 4-methyl-5-hexanolide (**17**) show characteristic mass spectra and occur as two closely eluting peaks: the two possible diastereomers. Aciphyllene (**19**) shows an identical mass spectrum and retention index to those reported in a critically evaluated data compilation [S1].

The compounds methyl methylsalicylate, methyl dimethylbenzoate (**24**), and methylbutenolide exhibited mass spectra similar to those in mass-spectral libraries, but no information of the retention indices of all possible isomers was available. Therefore, a full characterization was not possible. The mass spectrum of trimethyloxazole was identical to those in the databases, but again no retention-index information was found.

**Table S1:** Details of microorganisms used for the analysis of volatile compounds.

| No. | Species | Strain | Source |
|---|---|---|---|
| 1 | *Mtb* | H37Rv | Lab. strain |
| 2 | *Mtb* | H37Ra | Lab. strain |
| 3 | *Mtb* | Beijing 2 | Human-Netherlands |
| 4 | *Mtb* | Beijing 5 | Human-South Africa |
| 5 | *Mtb* | Beijing 6 | Human-Mongolia |
| 6 | *M. smegmatis* | $MC^2155$ | n/a |
| 7 | *M. avium* subspecies avium | n/a | n/a |
| 8 | *M. scrofulaceum* | n/a | n/a |
| 9 | *M. vaccae* | n/a | n/a |
| 10 | *M. aichiense* | LMG 19259 | Soil |
| 11 | *M. aurum* | LMG 19255 | Soil |
| 12 | *M. neoaurum* | LMG 19258 | Soil |
| 16 | *Nocardia asteroides* | LMG 4062 | n/a |
| 17 | *N. africana* | DSM 44499 | Human-Sudan |

## Media composition

### Middlebrook 7H9 Broth

Approximate formula per liter of purified water: monopotassium phosphate 2.0 g, disodium phosphate 1.5 g, monosodium glutamate 0.5 g, sodium citrate 0.1 g, ammonium sulfate 0.5 g, pyridoxine 0.001 g, zinc sulfate 0.001 g, copper sulfate 0.001 g, biotin 0.5 mg, calcium chloride 0.5 mg ferric ammonium citrate 0.04 g, magnesium sulfate 0.05 g,
ADC enrichment: sodium chloride 0.85 g, bovine albumin (fraction v) 5.0 g, dextrose 2.0 g, catalase 4.0 mg, sodium pyruvate 1.0 g

### Difco™ Mycobacteria 7H11 Agar

Approximate formula per 900 mL purified water:
Pancreatic digest of casein 1.0 g, L-glutamic acid 0.5 g, sodium citrate 0.4 g, pyridoxine 1.0 mg, biotin 0.5 mg, ferric ammonium citrate 0.04 g, ammonium sulfate 0.5 g, disodium phosphate 1.5 g, monopotassium phosphate 1.5 g, magnesium sulfate 0.05 g, agar 15.0 g, malachite green 1.0 mg
OADC enrichment: Approximate formula per liter purified water:
bovine albumin 50.0 g, dextrose 20.0 g, catalase 0.03 g, oleic acid 0.6 g

## References

[S1] Joulain, D.; König, W. A. *The Atlas of Spectral Data of Sesquiterpene Hydrocarbons*; E.-B. Verlag: Hamburg, 1998.
